# German translation of the Characterizing Freezing of gait questionnaire: implementation of the TRAPD process

**DOI:** 10.1186/s41687-025-00967-1

**Published:** 2025-11-14

**Authors:** Agnes Wilhelm, Jessie Janssen, Malena Teufelhart, Kaylena Ehgoetz Martens, Alice Nieuwboer, Peter Augat

**Affiliations:** 1 Institute Therapeutic and Midwifery Sciences, IMC Krems University of Applied Sciences, Krems an Der Donau, Austria; 2 Institute of Health Sciences, University of Applied Sciences St. Pölten, St. Pölten, Austria; 3 Department of Kinesiology and Health Sciences, University of Waterloo, Waterloo, Canada; 4 Department of Rehabilitation Science, KU Leuven, Leuven, Belgium; 5 Institute for Biomechanics BG Unfallklinik Murnau, Murnau Am Staffelsee, Germany; 6 Institute for Biomechanics Paracelsus Medical University Salzburg, Salzburg, Austria

**Keywords:** Freezing of gait, Parkinson’s Disease, Patient-reported outcome measurement, Screening, Subtypes, Translation

## Abstract

**Background:**

Freezing of Gait (FOG) is a severe symptom of Parkinson’s Disease (PD) that affects mobility and quality of life. The ‘Characterizing Freezing of Gait Questionnaire’ (C-FOG) is an assessment tool for screening and determining subtypes of FOG. However, it currently only exists in the English language. This study aimed to translate the C-FOG from English to German

**Methodology:**

This project was conducted from September 2024 to February 2025 at a University of Applied Sciences in Krems, Austria and applied the Translation, Review, Adjudication, Pretest, and Documentation (TRAPD) process. This included professional translations, 1 review-workshop with 4 participants, adjudication, pre-testing in 2 workshops with 11 participants, including 8 healthcare workers and 3 people with Parkinson’s disease, and parallel detailed documentation of all changes and decisions.

**Results:**

In the first translation stage of the C-FOG two professional translators provided two different preliminary German translations. The original questionnaire was divided into 62 translation units to provide a detailed descriptive analysis of the adaptations made in each step of the translation process. During the review stage, 31 units (50%) of the preliminary translations required adaptation. During the pre-test stage, the participants discussed the German translation of the C-FOG that had been adjudicated in the previous stage. This resulted in the need for further adaptations of 25 translation units (40%), while 37 units (60%) required no additional changes. Following back-translation by a professional translator and feedback from the first author of the original questionnaire, the German version of the C-FOG, the C-FOG-D, was finalized.

**Conclusion:**

This study provides a systematically conducted German translation of the Characterizing Freezing of Gait Questionnaire using the TRAPD process to ensure understandability and equivalence to the original questionnaire. A full consensus was reached among the participants involved in the final translation stage. For future validation studies and potential modifications to the questionnaire, it is necessary to include more people with PD experiencing FOG. A larger sample size could offer a more comprehensive perspective on how individuals differ in their perceptions of FOG, and to what extent the language used in the questionnaire effectively captures these experiences.

**Supplementary information:**

The online version contains supplementary material available at 10.1186/s41687-025-00967-1.

## Abstract (fr)

**Why is this study needed?:**

Freezing of Gait (FOG) is a serious symptom of Parkinson’s disease that affects mobility and quality of life. FOG is when someone suddenly feels like their feet are glued to the floor and can’t move forward, even though they want to walk. The Characterizing Freezing of Gait Questionnaire (C-FOG) is a way to identify and measure different types of FOG.

**What is the key problem/issue/question this manuscript addresses?:**

The Characterizing Freezing of Gait Questionnaire, that assesses this symptom, is only available in English and therefore the use in German-speaking countries is limited.

**What is the main point of your study?:**

This study wanted to provide an easy-to-understand and accurate translation of the Characterizing Freezing of Gait questionnaire from English to German, so that it can be used in German-speaking countries.

**What are your main results and what do they mean?:**

This study provides a German translation of the C-FOG using a step-by-step process to make sure it is easy to understand and the same as the original questionnaire. Everyone on the translation project team agreed to this final German version.

## Background

Parkinson’s Disease (PD) is the second most common neurodegenerative disease. In 2022, PD affected approximately 295,000 individuals in Germany, and 16,000 in Austria [1, 2]. Freezing of Gait (FOG) is a disabling symptom of PD and is defined as an episodic absence or reduced forward movement of the feet despite the intention to walk [3, 4]. The Characterizing Freezing of Gait Questionnaire (C-FOG) was developed as a tool for screening different aspects of FOG, particularly providing a detailed screening of the triggering factors [5]. According to a previous cluster analysis by Ehgoetz Martens et al. [5], people with FOG were categorized into three main subtypes: asymmetric-motor, sensory-attention and anxious FOG. Whilst asymmetric-motor FOG is worsened by environments such as turning that requires asymmetrical stepping, sensory-attention FOG was worsened by environments that demand heightened attention such as clutter or the dark. Anxious FOG was exacerbated by stress and emotional factors [5].

In addition to the FOG items from the Movement Disorder Society - Unified Parkinson’s Disease Rating Scale [6], there are two validated questionnaires for assessing FOG: the original Freezing of Gait Questionnaire (FOGQ) [7] and the New Freezing of Gait Questionnaire (NFOGQ) [8]. Both questionnaires evaluate FOG severity based on frequency, intensity, duration of the longest episodes, and its impact on quality of life and daily activities [9, 10]. The NFOGQ focuses only on gait initiation and turning, as those are the most common FOG triggers [11]. However, none of these tools provide questions on the different non-motor triggers of FOG, as experienced in daily life. The C-FOG includes common triggers as well as relief-strategies for FOG to evaluate whether patterns exist amongst this heterogeneity to distinguish between the various subtypes described. Moreover, the C-FOG has standardized questions about freezing of speech, freezing of the arms and legs while not walking. These non-gait freezing phenomena or freezing-like motor blocks possibly share similar mechanisms as FOG [12].

The C-FOG is a relatively new assessment tool but is already recognized in scientific literature [13]. While information on the use in clinical practice is limited and there is no clear evidence yet of its impact in English-speaking countries, its international relevance is growing, as shown by a recent Japanese translation in 2021 [14]. To the best of our knowledge, no German-language equivalent of the C-FOG questionnaire exists. Thus, the lack of a German translation limits the accessibility of the C-FOG and restricts its applicability in the DACH region (Germany, Austria and Switzerland). The aim of this project was to develop a German translation of the C-FOG questionnaire to enable its application in German-speaking populations.

## Methods

To translate the C-FOG from English to German a five-stage process based on the TRAPD process was applied involving professional translation, expert review, adjudication, pre-testing and parallel thorough documentation of all changes and decisions [15]. According to the Cross-Cultural Survey Guidelines [15], the TRAPD process is one approach for translating and adapting questionnaires. This method was employed as it is a team-based, iterative approach that emphasizes collaboration among people with diverse expertise, allowing for both linguistic accuracy and contextual relevance [16]. Other established frameworks exist, such as the guidelines proposed by Wild et al. [17] and the recommendations developed by the ISOQOL Translation and Cultural Adaptation Special Interest Group [18]. All frameworks include a structured process for translation, such as initial translation, expert review and adaptation, and cognitive debriefing or pre-testing with target populations [16–18]. But as interprofessional collaboration was seen as important for the translation of this complex phenomenon, the TRAPD process was followed in this project.

The translation was carried out from September 2024 to February 2025 at the IMC Krems, University of Applied Sciences in Austria.

The English version of the C-FOG questionnaire was provided in the original publication [19]. Permission to translate was granted by the first author of the questionnaire, who was also available for feedback in the translation process.

The final translated German version of the C-FOG will be called ‘Charakterisierung von Freezing of Gait – Deutsche Version’ (C-FOG-D).

### Original source text

The C-FOG questionnaire is a 35-item self-assessment tool developed with input from patients with PD, caregivers, and researchers. The C-FOG showed good reliability and validity, with severity scores correlating moderately with actual time spent frozen (*r* = 0.54) and strong internal consistency (Cronbach’s α = 0.937) [5]. These results suggest that it effectively captures freezing patterns linked to specific environmental triggers that vary across individuals [5].

The C-FOG is organized into four sections: Section I (Severity) focuses on assessing the presence, severity, and treatment responsiveness of freezing of gait. Section II (Triggers) examines situational factors that provoke FOG. Section III (Strategies) evaluates the effectiveness of commonly used strategies to reduce or overcome FOG and Section IV (Other Types of Freezing) addresses the presence and severity of other forms of freezing, such as upper limb freezing. To prepare for the workshops, the questionnaire was divided into translation units to support descriptive analysis by the lead researcher (AW). To avoid confusion with the term ‘items’, which typically refer to individual questions and answers in a questionnaire, the term ‘translation unit’ was used to describe manageable segments of text that supported a coherent translation process [20]. These units included different categories: 2 administrative items (date and name), 1 title, 7 instructions, 15 questions, and 37 answer options, totaling 62 translation units. In Sections I (‘Severity’) and IV (‘Other Types of Freezing’), each question and its answer options were grouped together and counted as a single unit. In contrast, in Sections II (‘Triggers’) and III (‘Strategies’), questions and answer options were counted separately.

The original source provides the C-FOG as the main questionnaire, accompanied by an additional ‘Cluster Solution for Section II Items’. This supplementary component includes 12 answer options that are identical with those presented in Section II of the main questionnaire. One title and three headlines that were not used in Sections I to IV of the main questionnaire were descriptively analyzed throughout the translation process.

### Translation process

**Fig. 1:**
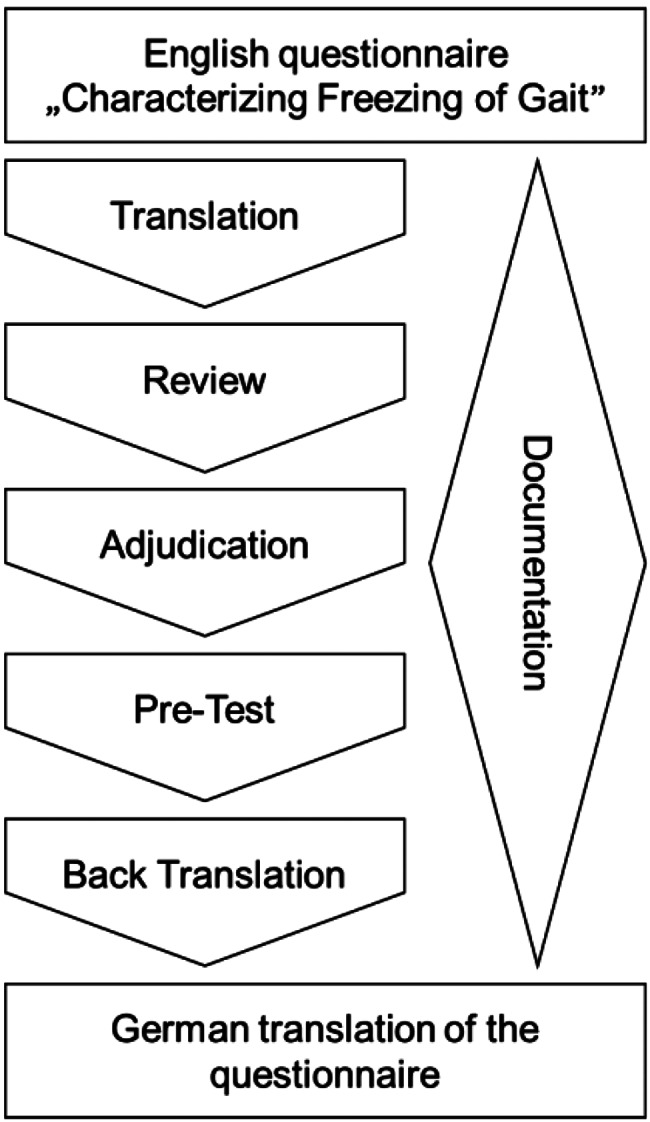
Visualization of the TRAPD process including back translation

In the following the five stages of the TRAPD process [15] (see Fig. 1) will be explained. For consensus a minimum of 75% agreement [21] was set up as a threshold in all translation stages.

Translation: The C-FOG and the ‘Cluster Solution for Section II Items’ were translated into German by two professional translators contracted through a translation bureau situated in Vienna. The translators all had German as their first language and are certified translators for English and German.

Review: The review team (*n* = 4) focused on comparing and discussing the two translations provided by the professional translators. The C-FOG was discussed and analyzed regarding understandability and equivalence to the original questionnaire, focusing on linguistic issues from a theoretical perspective. The goal was to reach consensus for each translation unit on necessary adaptations and to decide if specific units required further clarification during the pre-testing phase or by the first author of the original questionnaire.

Adjudication: The lead researcher (AW) compiled an adjudicated German version based on the outcomes of the review stage and double-checked this version with the review-team for consensus.

Pre-testing: During the pre-test workshops, the adjudicated German translation of the C-FOG from the review stage was thoroughly discussed. The primary focus was on the understandability of each unit from an applied perspective, involving members of the target population, such as diverse healthcare professionals and people with PD. Participants were asked to explain how they would respond to the questions and to describe how alternative wording might influence their answers. This allowed insights into how members of the target population interpret and respond to the translated items. For selected units the original English phrasing was provided by the lead researcher to ensure equivalence. The questions from the review team and the answers given by the first author of the questionnaire, via e-mail, were also presented to the pre-test team. One 3-hour pre-test workshop was initially planned. However, as the discussions were lengthier than anticipated, and only half of the translation units were discussed at that time, a second 3-hour workshop was scheduled four weeks later to continue the discussion of the remaining translation units. In both workshops diverse healthcare professionals and people with PD participated. Based on the feedback of the participants, translation units were either accepted in their current form or adapted after consensus. The lead researcher (AW) compiled an adjudicated version and double-checked it via e-mail with the pre-test-team.

Back-translation: The questionnaire was subsequently back translated from German to English by the same translation bureau situated in Vienna that handled the initial translations. This task was carried out by different professional translators who had English as their first language and were not familiar with the original English version. The back-translation was then sent to the first author of the English C-FOG questionnaire for feedback.

Documentation: The entire translation process, including all decisions, rationales, and changes made at each stage, was thoroughly documented by the lead researcher (AW) and double checked with the workshop teams in the respective translation stage for consensus.

### Participants

#### Recruitment review workshop

The recruitment process for the review workshop involved sending an invitation via an internal communication system at the University of Applied Sciences, addressing both students and employees. Interested individuals accessed an online portal to review study details, self-assess their C1 language skills, and verify eligibility based on the inclusion and exclusion criteria. Nine individuals met the criteria and actively enrolled. Inclusion criteria were: student or employee at the University of Applied Sciences; over 18 years old; self-assessed C1-level proficiency in English and German, based on a customized ‘yes or no’ dichotomous questionnaire aligned with the Common European Framework of Reference for Languages level C1 [22], covering oral production, reading comprehension, and cooperation. This questionnaire was designed for the recruitment process by the lead researcher (AW). Oral production was evaluated by the participant’s ability to explain complex topics clearly and express themselves fluently. Reading comprehension focused on understanding complex text and reading a variety of materials with ease. Cooperation assessed the participants’ ability to engage in discussions and integrate ideas smoothly with others (see full questionnaire in the supplementary material). Participants were eligible if they scored 4 or more points with a maximum score of 6 points (≥ 67%). Participants’ first language had to be German or English, defined as a language acquired unconsciously in early childhood, typically before the age of three [23]. The date was determined by the project lead’s availability and selected to maximize participant attendance. Of the nine eligible individuals, only the four who were available on that day could participate in the workshop.

Four university employees (self-reported gender: two males, two females) participated in a one 3-hour review workshop. Two people were in the age group 31–40 years, one person in the age group 41–50 years and one person in the age group 51–60 years; German was the first language for three people, and English for one. In the self-designed questionnaire, two participants assigned a rating of 6 out of 6 points (100%) to their language skills in the customized questions regarding oral production, reading comprehension, and cooperation in both languages. Two participants rated their skills at 6 out of 6 points (100%) in their respective first language (one person English, one person German) and 4 out of 6 points (67%) in the other. The participants had diverse occupations, namely car mechanic, occupational therapist, physiotherapist and nurse. Every participant worked with German and English texts in their everyday life.

#### Recruitment pre-test workshops

The pre-test workshops focused on the adjudicated German translation from the review workshop. A convenience sample was recruited via personal emails sent within the Department of Health Sciences at the University of Applied Sciences. The aim of recruitment was to acquire at least one person from every healthcare profession within a study program at the university where the project was conducted, to ensure diverse perspectives. Three individuals with Parkinson’s disease (PD) were invited directly via email, as they were already involved in a separate Patient and Public Involvement project at the University of Applied Sciences. Fluency in German was required but was not formally assessed.

One person from the review workshop also participated in both pre-test workshops. The pre-test workshops included ten and eight participants, respectively. Overall, 11 individuals participated in the pre-test workshops. Three participants took part in the first pre-test workshop but did not attend the second, while one person participated only in the second pre-test workshop. Participants in both workshops represented people with PD and a diverse range of healthcare professionals, including physiotherapists, occupational therapists, a nurse, a midwife, a music therapist, and a medical doctor. Their self-reported gender was three males and eight females. One person was in the age group 18–30, three people in the age group 31–40, three people in the age group 41–50, one person in the age group 51–60 and three people in the age group ≥ 61. Among the participants, three individuals with PD were included. All three attended the first pre-test workshop, and two of them also participated in the second. No demographic table is included in this report to protect participant confidentiality. Due to the specific nature of the collected information, there is a risk that individuals could be identified if demographic details were disclosed.

## Results

### Translation

In the two preliminary professional German translations of the questionnaire 17 out of 62 translation units (27.4%) were identical (n = 2 administrative items, n = 3 instructions and n = 12 answer options), while 45 units (72.6%) showed unspecified differences (n = 1 title, n = 4 instructions, n = 15 questions and n = 25 answer options). In the ‘Cluster Solution for Section II Items’ 2 units (50%) were identical (n = 2 headlines) and 2 units (50%) were different (n = 1 title, n = 1 headline).

### Review

**Fig. 2:**
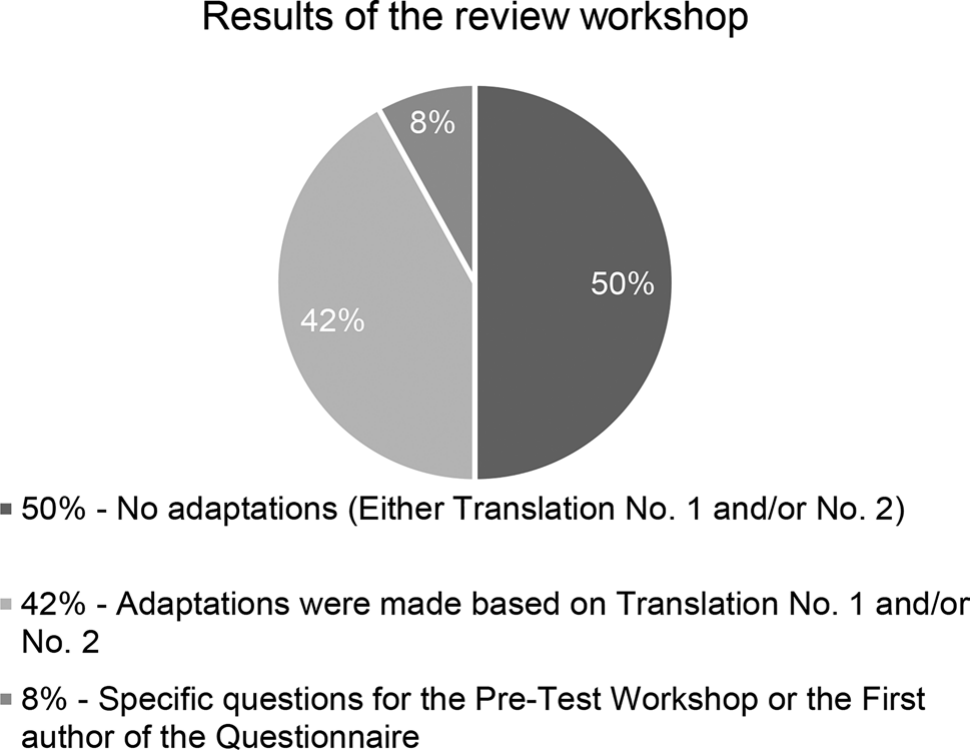
Overview of the results of the review workshop

In the first stage of the review workshop, in 31 out of 62 translation units (50%) a consensus was reached on either one of the translations or on both, if they were identical. In 26 units (42%) adaptations were made based on translation No. 1 and/or No. 2 (see example in Table 1). In 5 units (8%) the review team had specific questions for the pre-test team and/or the first author of the questionnaire (see Fig. 2). All decisions reached a 100% consensus among the participants.

**Table 1:** Review stage: category ‘adaptations were made based on translation No. 1 and/or No. 2’

Translation stage	Review stage
Translation unit category	Instructions
English original	This questionnaire will ask you questions about Freezing of Gait, […] For the first section please circle the appropriate answers.
Translation No. 1/Translation No. 2	In diesem Fragebogen werden Ihnen Fragen zu Bewegungsblockaden beim Gehen gestellt. […] Bitte kreisen Sie im ersten Abschnitt die entsprechenden Antworten ein./In diesem Fragebogen werden Ihnen Fragen zum Phänomen ‘Freezing of Gait’ (FOG) gestellt. […] Bitte kreisen Sie im ersten Abschnitt die zutreffenden Antworten ein.
German translation after Review workshop	In diesem Fragebogen werden Ihnen Fragen zu Freezing of Gait (Bewegungsblockaden beim Gehen) gestellt. […] Bitte kreuzen Sie im ersten Abschnitt die entsprechenden Antworten an.
Consensus/Adaptation/Discussion	100% consensus Based on version 1; Adaptation: ‘To tick the appropriate answer’ instead of ‘to circle the appropriate answer’. Discussion: Ticking boxes is more common in German questionnaires than circling the answers;

### Adjudication

In the subsequent adjudication process, all adaptations that were agreed upon by consensus during the review workshop were integrated into a consolidated joint German translation by the lead researcher (AW). This adjudicated translation was sent out to the participants of the review team and resulted in full consensus (100%) of all 62 translation units and no further modifications to the translation process had to be made.

### Pre-testing

**Fig. 3:**
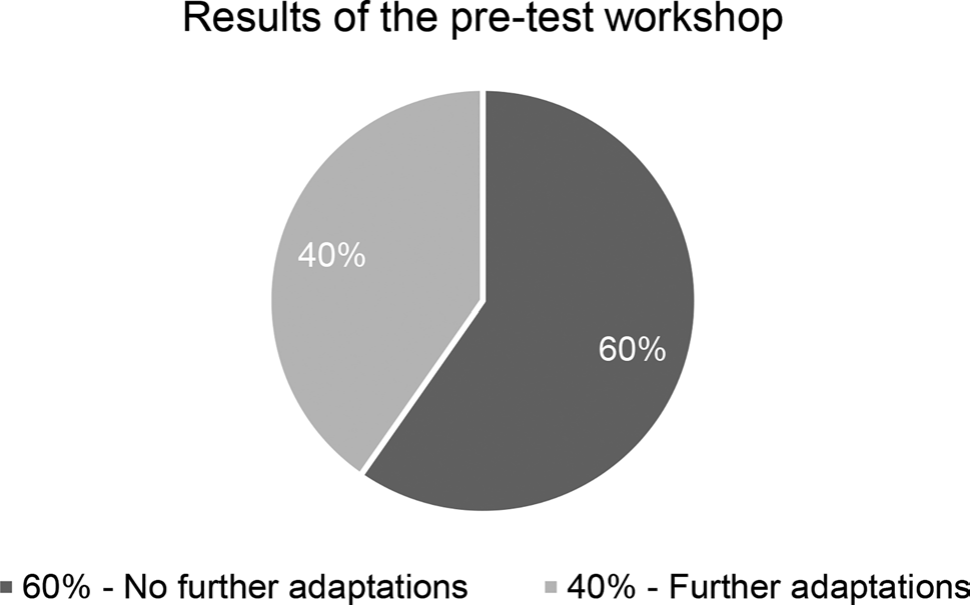
Overview of the results of the pre-test workshop

In the pre-test stage, translation units were reviewed based on the German version of the C-FOG translation from the adjudication stage. These translation units were either accepted in their current form or adapted. The pre-test workshops resulted in further adaptations of 25 out of 62 units (40%) (example see Table 2), while 37 units (60%) required no additional changes (see Fig. 3). 100% consensus in the pre-test team was reached in 61 units (98,4%). In 1 unit (1,6%), an 87,5% consensus was reached.

**Table 2:** Pre-test stage: category ‘further adaptations’ during the pre-test stage

Translation stage	Pre-test stage
Translation unit category	Question
English original	Have you ever experienced freezing in your legs/feet when you were NOT walking (e.g. wiping your feet, to draw your chair up to the table, etc.)
German version after review-workshop	Haben Sie schon einmal Freezing in den Beinen/Füßen erlebt, wenn Sie gerade NICHT gehen (z. B. Wenn Sie Ihre Schuhe abstreifen/abputzen, wenn Sie Ihren Stuhl zum Tisch rücken usw.)?
Consensus German translation after pre-test workshop	Haben Sie schon einmal Freezing in den Beinen/Füßen erlebt, wenn Sie gerade NICHT gehen (z. B. wenn Sie Ihre Schuhe abstreifen/auf der Fußmatte abputzen, wenn Sie mit Ihrem Stuhl zum Tisch rücken usw.)?
Consensus	100% Consensus 1. ‘Auf der Fußmatte abputzen’ instead of ‘abstreifen’; 2. ‘mit’ added
Discussion	1. ‘Abstreifen’ could also mean taking off one’s shoes. ‘Auf der Fußmatte’ added to clearly emphasize the meaning; 2. ‘mit’ added to reinforce the meaning of sitting on the chair while doing so

Inconsistencies in the source questionnaire came up during the pre-testing workshops (example see Table 3). No modifications were made, as the project’s scope was solely the translation of the questionnaire, but the discussions were documented.

**Table 3:** Pre-test stage: documented inconsistency in the source questionnaire

Item 1.1. How often do you experience Freezing of Gait? Once/year; Once/month; Once/week; Once/day; More than once/day;
Item 1.2 If you answered, ‘More than once/day’. On average, how many Freezing of Gait episodes do you experience on a daily basis? N/A; 1–2; 3–5; 6–10; 11–20; > 20
Discussion: About the necessity of the answer option ‘N/A’ if this question is only applicable when participants have answered question Item 1.1 with ‘yes.’ Discussion conveyed that it would change the questionnaire too much if this answer option were left out, and that in the population of people with PD, it could be that they can’t answer this question in detail, so a ‘I don’t know’ instead of an N/A answer could be a good alternative option.

Following the second pre-test workshop, the lead researcher (AW) incorporated all adaptations of Section I to IV of the C-FOG questionnaire. These were then subjected to a feedback loop within the pre-test team via email for gathering feedback and final confirmation. After a review phase of two weeks, the final version was accepted with a 100% consensus.

### Back translation

The back translation was provided by professional translators who were native English speakers and were located at the same translation bureau where the initial translation was conducted. None of them were familiar with the original source material. It was sent to the first author of the original questionnaire for feedback. No further changes were required.

The four translation units of the ‘Cluster Solution for Section II Items’ did not undergo a formal consensus process or feedback loops, as they are solely headings for the cluster analysis for the person evaluating the questionnaire. These headings are not questionnaire items themselves. The actual questionnaire items, which are identical in both the main instrument and the additional ‘Cluster Solution for Section II Items’ section, were fully included in the translation process. The translations of the four units were based on the professional translations from the first translation stage, with the lead researcher (AW) making the final decisions.

The format design of the C-FOG-D was conducted by the lead researcher (AW), with non-formal feedback loops with employees in the Department of Health Sciences. The format was changed from the original English version because the German translations were mostly longer and to maintain the four-page layout with one section per page. The C-FOG-D was sent via e-mail to the three participants with PD for feedback. Following confirmative feedback from all involved, the format design of the C-FOG-D was finalized.

## Discussion

This study describes the translation of the C-FOG from English to German using the TRAPD process, resulting in the German version C-FOG-D.

A review and two pre-test workshops were held in this process. The participants in all workshops were tasked with focusing on understandability and equivalence to the original questionnaire. As the detailed discussions and adaptations made during the process were documented, trends emerged regarding different categories of reasons for those adaptations. These changes could be linked to five aspects of linguistics. Semantic equivalence, meaning, that two sentences are a direct translation and have the same meaning in both languages [24]. Pragmatic equivalence, would be if both sentences would play the same role in both languages depending on the speaker, the addressee and the context of utterance [24]. Cultural equivalence preserves the cultural impact and the cultural context of the source text [25]. Syntax, is the arrangement of words and builds the sentence structure that is relevant to the meaning [26]. Text and format structure refers to the organization and visual layout of the text [27]. Linguistically it could be interesting to further analyze the nature of the changes in depth.

Two examples of the category ‘pragmatic equivalence’ show the need for specific phrasing regarding FOG. The examples are the translations of ‘when you have to take the first step after standing up’ and ‘feeling “glued” to the floor’. In German, ‘standing up’ would be translated as ‘aufstehen’, but this could also be translated back as ‘getting up’, as in ‘rising from bed after sleeping’. Therefore, to convey the intended meaning, ‘from a sitting position’ (‘aus dem Sitzen’) was added to clarify the sequence of movements from sitting to standing. Those two different meanings could change the outcome of the questionnaire regarding FOG, as it could make a difference if the person experiences FOG while getting up or standing up. As for the translation of ‘feeling “glued” to the floor’, the participants chose to provide two translation options: ‘fest zu kleben/festgeklebt zu sein’. The discussion conveyed that offering both options makes it possible to express being firmly glued to the floor ‘from the inside’ or ‘from the outside’. This shows the subjective variability in how people with PD might experience FOG. Either as being glued ‘from the inside’ as an internal block or as being glued ‘from the outside’ like an external restraint.

The following example illustrates the category of ‘Cultural Equivalence’: The employment of divergent strategies to overcome FOG is potentially depending on factors such as country, geographical location, the workplace or personal experience. The act of ‘stepping over […] a string attached to my walking stick’ was not a known strategy among the participants of both workshops during the translation process. The phrase was modified to ‘Stepping over […] an object attached to a walking stick’ to allow for individual interpretation, while maintaining the same mode of action as a cueing mechanism. The participants indicated a preference for the term ‘walking aid’, however, this was deemed to be too divergent from the original questionnaire.

In our study 17 translation units (27.4%) of the two professional preliminary translations were identical and 45 units (72.6%) showed differences. These results are comparable to the findings in other previous studies. Naber et al. [28] for example reported that in their study there was complete similarity between both preliminary translations in 31.7% of translation units; in 41.5%, there was partial similarity; and in 26.8%, there was minimal or no similarity. The minimal differences typically stemmed from different sentence structures, while cases of no similarity were due to different word choices [28]. In our study we didn’t categorize the differences in partial or minimal/no similarity.

Other studies also have quantified their translation processes. In one study for example, that also used the TRAPD process, 26% of the initial translation units needed a further complex adaptation and 36% of the units needed cultural adaptations. This amounted to a total of 62% of the adaptations [29]. In the present study 50% of the professional translations required some form of adaptation in the initial stage. Depending on the different pre-specified translation units for the analysis, these numbers could vary. In this present study the translation units were based on the items of the questionnaire, as they formed a set and belonged together in this context.

In parallel with this methodological linguistic approach FOGs variability poses challenges for both its definition and measurement [30]. The recent literature provides different hypotheses for the occurrence of FOG. The C-FOG describes three different subtypes whereas in other scientific work, up to four or five underlying hypotheses are described. The C-FOG attempts to provide an assessment to screen FOG subtypes in a clinical setting, forming the basis to connect the results with other mobility or gait assessments and to explore the possibility of determining the existence, distribution and differentiation of those subtypes.

The use of the C-FOG-D in the DACH region could be a first step to support German speaking healthcare providers, like physiotherapists, to screen different subtypes using a questionnaire in a clinical setting. Based on the results, targeted interventions that address the specific needs of individuals with different FOG subtypes could be developed. A systematic review by Gilat et al. [31] showed that general fitness and health exercises had no impact on FOG. In contrast, specific FOG prevention measures, such as the use of cueing strategies or learning FOG prevention strategies for home use, significantly reduced the intensity of FOG. So specific intervention approaches are needed to treat FOG.

Additionally, the C-FOG has standardized questions about non-gait freezing phenomena or freezing-like motor blocks like freezing of speech, freezing of arms and freezing of legs while not walking [5]. To our knowledge no other FOG specific questionnaire includes those phenomena. The screening for non-gait freezing phenomena with the C-FOG-D would allow German speaking health care providers to assess those symptoms as well and potentially include them in their intervention plan.

## Strengths and limitations

In comparison to the original TRAPD process [15] this study included not only people with PD in the pre-test stage, but different health care providers to incorporate diverse perceptions and a broader applicability, as different backgrounds and perspectives influence how people interpret and translate text [32]. In the pre-test stage a convenience sample was used, and FOG was neither an inclusion criterion, nor was it specifically assessed. None of the participants with PD indicated that they experienced FOG themselves, but they could contribute relevant insights based on acquaintances with the condition. For the scope of this study, regarding the translation and not the modification of the questionnaire, the participants provided valuable insights from the perspective of the target audience, which is people with PD experiencing FOG. For future studies and possible modifications of the questionnaire, participants with PD from all DACH countries should be included. Additionally, a larger sample size is recommended, as pre-testing with 15–30 participants is advised to ensure validity [33]. As mentioned before, FOG is a highly heterogeneous phenomenon, varying in frequency, severity and triggers [30]. Expanding the sample size could provide broader insights into an individual’s perception of FOG and the questionnaire’s effectiveness to capture these experiences through its linguistic phrasing. It would also enable subgroup analyses to explore the questionnaire’s performance across different clinical profiles.

Another challenge in this study was, the pluricentric nature of the German language, meaning that it has multiple standard forms across different countries [34]. Austria, Germany, and Switzerland each have their own standard varieties of German, which are recognized and used officially within their respective borders. These variations are comparable to distinctions among British, American, and Australian English [35]. For this translation the lead researcher (AW), in accordance with the review and the pre-test team, decided during the process to prefer ‘German words’ to ‘Austrian words’ to reach better and broader understandability in all German speaking countries of the DACH region.

## Conclusion

The German translation of the Characterizing Freezing of Gait Questionnaire (C-FOG-D) was developed using the TRAPD process to ensure understandability and equivalence to the original version. The final version of the C-FOG-D was approved with full consensus by all eleven participants of the pre-test workshop including healthcare professionals and individuals with PD. In future studies the C-FOG-D needs to be validated in a larger sample of individuals with PD experiencing FOG, across German speaking countries in the DACH region. Furthermore, future cross-cultural studies need to explore the distribution of the FOG subtypes and the potential of the C-FOG-D to differentiate between them.

## Electronic supplementary material

Below is the link to the electronic supplementary material.

Supplementary Material 1 - C-FOG-D including the ’Cluster Solution for Section II Items’

Supplementary Material 2 - C-FOG- D / C1 Self-Assessment

## Data Availability

The data used for this study, including the documentation and results of the review workshop and the pre-test workshop, can be made available on a secure server by the corresponding author on reasonable request.

## Abbreviations

C-FOG: Characterizing Freezing of Gait Questionnaire
C-FOG-D: Charakterisierung von Freezing of Gait – Deutsche Version
FOG: Freezing of Gait
FOGQ: Freezing of Gait Questionnaire
NFOGQ: New Freezing of Gait Questionnaire
NÖGUS: Niederösterreichischer Gesundheits- und Sozialfonds
PD: Parkinson’s Disease
TRAPD: Translation, Review, Adjudication, Pretesting, and Documentation
