# Supplementary material for: German translation of the Characterizing Freezing of gait questionnaire: implementation of the TRAPD process
Source: J Patient Rep Outcomes. 2025 Nov 14;9:132. doi: 10.1186/s41687-025-00967-1 (PMC12618777; doi:10.1186/s41687-025-00967-1)
Supplement: Supplementary file 1 — Supplementary Material 1 - C-FOG-D including the ’Cluster Solution for Section II Items’ [file 41687_2025_967_MOESM3_ESM.pdf]

Name:

Datum:

Charakterisierung von Freezing of Gait

In diesem Fragebogen werden Ihnen Fragen zu Freezing of Gait (Bewegungsblockaden beim Gehen) gestellt. Dabei geht es darum, dass Sie bei den einzelnen Ereignissen plötzlich nicht mehr in der Lage sind, Ihre Füße beim Gehen vorwärts zu bewegen, und Sie das Gefühl haben, am Boden „fest zu kleben“/„festgeklebt zu sein“.

Bitte kreuzen Sie im ersten Abschnitt die entsprechenden Antworten an.

1.0 Haben Sie jemals Freezing of Gait erlebt?

◻ Ja	◻ Nein

1.1. Wenn Sie oben mit „Ja“ geantwortet haben, beantworten Sie bitte die folgenden Fragen:
Wie oft kommt es bei Ihnen zu Freezing of Gait?

◻ Einmal im Jahr	◻ Einmal im Monat	◻ Einmal in der Woche
◻ Einmal am Tag	◻ Mehr als einmal am Tag	

1.2. Wenn Sie „Mehr als einmal am Tag“ geantwortet haben:
Wie oft am Tag kommt es bei Ihnen im Durchschnitt zu Freezing of Gait?

◻ Nicht zutreffend	◻ 1- bis 2-mal	◻ 3- bis 5-mal
◻ 6- bis 10-mal	◻ 11- bis 20-mal	◻ >20-mal

2.0 Wie lange dauert Ihr Freezing of Gait normalerweise an?

◻ 1-2 Sekunden	◻ 3-5 Sekunden	◻ 6-10 Sekunden
◻ 11-15 Sekunden	◻ 16-20 Sekunden	◻ >20 Sekunden

3.0 Zu welcher Tageszeit kommt es bei Ihnen am häufigsten zu Freezing of Gait?

◻ Morgens	◻ Mittags	◻ Abends
◻ Nachts	◻ Kein Unterschied	

4.0 Wie verändert sich Ihr Erleben von Freezing of Gait durch die Einnahme Ihrer Medikamente?

◻ Verbessert sich	◻ Verschlechtert sich
◻ Keine Veränderung	◻ Ich nehme keine Medikamente

5.0 Welchen Einfluss hat die Tiefe Hirnstimulation auf das Ausmaß von Freezing of Gait, das Sie erfahren?

◻ Verbessert sich	◻ Verschlechtert sich
◻ Keine Veränderung	◻ Ich habe keine Tiefe Hirnstimulation

Name:

Datum:

Im folgenden Abschnitt fragen wir Sie nach bestimmten Auslösern, die zu Freezing of Gait führen können. Bitte kreuzen Sie bei jeder Frage die für Sie entsprechende Zahl an. Damit geben Sie an, wie häufig dieser Auslöser bei Ihnen zu Freezing of Gait führt. (Clusterlösung im Anhang Seite I)

**Auslöser**	**Niemals**	**Selten**	**Manchmal**	**Oft**	**Immer**
1. Wenn Sie sich auf der Stelle drehen (z.B. Umdrehen in der Küche).	0	1	2	3	4
2. Wenn Sie um eine Ecke gehen.	0	1	2	3	4
3. Wenn Sie beim Gehen sprechen.	0	1	2	3	4
4. Wenn Sie von jemandem oder von etwas abgelenkt werden.	0	1	2	3	4
5. Wenn Sie hektisch sind oder sich beeilen.	0	1	2	3	4
6. Wenn Sie sich ängstlich fühlen.	0	1	2	3	4
7. Wenn Sie durch eine Tür gehen.	0	1	2	3	4
8. Wenn Sie in einer vollgestellten bzw. überladenen Umgebung sind.	0	1	2	3	4
9. Wenn Sie nach dem Aufstehen den ersten Schritt machen müssen (z.B. aus dem Sitzen).	0	1	2	3	4
10. Wenn Sie nach einer Drehung im Stand den ersten Schritt machen müssen.	0	1	2	3	4
11. Wenn Sie im Dunkeln gehen.	0	1	2	3	4
12. Wenn Sie eine Steigung hinauf gehen.	0	1	2	3	4

Nennen Sie bitte weitere Situationen, in denen es üblicherweise bei Ihnen zu Freezing of Gait kommt und die oben nicht aufgelistet sind:

Name:

Datum:

Im nächsten Abschnitt sollen Sie die Wirksamkeit bestimmter Strategien bewerten, die zur Milderung von Freezing of Gait angewendet werden können. Bitte kreuzen Sie bei jeder Frage die entsprechende Zahl an. Damit geben Sie an, wie wirksam diese Strategie für Sie ist.

Niemals - Ich habe diese Strategie noch nie angewandt.
Selten - Diese Strategie ist selten wirksam, wenn ich Freezing of Gait überwinden möchte.
Manchmal - Diese Strategie ist manchmal (aber nicht immer) wirksam.
Oft - Diese Strategie hilft fast immer, wenn ich Freezing of Gait überwinden möchte.
Immer - Diese Strategie verhindert vollständig, dass ich Freezing of Gait erlebe.

**Strategie**	**Niemals**	**Selten**	**Manchmal**	**Oft**	**Immer**
1. Ein Lied singen.	0	1	2	3	4
2. Ein paar tiefe Atemzüge nehmen.	0	1	2	3	4
3. Auf ein Ziel konzentrieren (z.B. Linien auf dem Boden).	0	1	2	3	4
4. Gegen einen Ball treten, der an einer Schnur hängt.	0	1	2	3	4
5. Sich auf etwas anderes als die eigenen Füße konzentrieren (z.B. auf die Atmung).	0	1	2	3	4
6. So tun, als würde man Treppen steigen (z.B. Knie hochheben).	0	1	2	3	4
7. Über die Füße einer anderen Person oder über einen, am Gehstock befestigten Gegenstand, steigen.	0	1	2	3	4
8. Zählen (z.B. „1-2-3“ oder „Links-Rechts-Links-Rechts“ oder „Auf die Plätze, fertig, los!“).	0	1	2	3	4
9. Einen auf den Boden projizierten Laserstrahl verwenden.	0	1	2	3	4
10. Zum Takt eines Metronoms gehen.	0	1	2	3	4

Nennen Sie bitte alle weiteren Strategien, die Sie normalerweise zum Überwinden von Freezing of Gait verwenden, die oben nicht aufgelistet sind:

Name:

Datum:

Im folgenden Abschnitt werden Ihnen Fragen zu anderen Arten von Freezing gestellt, die auftreten können. Bitte kreuzen Sie die zutreffenden Antworten an.

1.0 Haben Sie schon einmal Freezing beim Sprechen erlebt (sind Sie z.B. beim Sprechen plötzlich ins Stocken geraten oder haben kein Wort herausgebracht, obwohl Sie wussten, was Sie sagen wollen)?

□ Ja	□ Nein

1.1 Wenn ja, wie häufig kommt das ungefähr vor?

□ Selten	□ Manchmal
□ Regelmäßig	□ Oft

2.0 Haben Sie schon einmal Freezing in Ihren Armen/Händen erlebt (z.B. beim Versuch, Ihre Post aus dem Briefkasten zu holen, beim Schreiben, bei der Benutzung des Computers usw.)?

□ Ja	□ Nein

2.1 Wenn ja, wie häufig kommt das ungefähr vor?

□ Selten	□ Manchmal
□ Regelmäßig	□ Oft

3.0 Haben Sie schon einmal Freezing in den Beinen/Füßen erlebt, wenn Sie gerade NICHT gehen (z. B. wenn Sie Ihre Schuhe auf der Fußmatte abputzen, wenn Sie mit Ihrem Stuhl zum Tisch rücken usw.)?

□ Ja	□ Nein

3.1 Wenn ja, wie häufig kommt das ungefähr vor?

□ Selten	□ Manchmal
□ Regelmäßig	□ Oft

Gibt es Situationen oder Auslöser, die bei Ihnen die oben angeführten Arten von Freezing verursachen? Wenn ja, geben Sie diese bitte unten an:

Sie haben den Fragebogen fertig ausgefüllt. Vielen Dank!

Name:

Datum:

Clusterlösung für Items aus dem Abschnitt „Auslöser“ auf Seite 2

Item	Auslöser	Cluster
1	Wenn Sie sich auf der Stelle drehen (z.B. Umdrehen in der Küche).	1
2	Wenn Sie um eine Ecke gehen.	1
7	Wenn Sie durch eine Tür gehen.	1
9	Wenn Sie nach dem Aufstehen den ersten Schritt machen müssen (z.B. aus dem Sitzen).	1
10	Wenn Sie nach einer Drehung im Stand den ersten Schritt machen müssen.	1
3	Wenn Sie beim Gehen sprechen.	2
4	Wenn Sie von jemandem oder von etwas abgelenkt werden.	2
5	Wenn Sie hektisch sind oder sich beeilen.	2
6	Wenn Sie sich ängstlich fühlen.	2
8	Wenn Sie in einer vollgestellten bzw. überladenen Umgebung sind.	3
11	Wenn Sie im Dunkeln gehen.	3
12	Wenn Sie eine Steigung hinauf gehen.	3

Freezing of Gait subtypes based on the data-driven approach by Ehgoetz Martens et al. (2018).

Cluster 1 = motor-asymmetry

Cluster 2 = anxiety

Cluster 3 = sensory-attention

Ehgoetz Martens, K. A., Shine, J. M., Walton, C. C., Georgiades, M. J., Gilat, M., Hall, J. M., Muller, A. J., Szeto, J. Y. Y., & Lewis, S. J. G. (2018). Evidence for subtypes of freezing of gait in Parkinson's disease. Mov Disord, 33(7), 1174-1178. https://doi.org/10.1002/mds.27417
