# Supplementary material for: German translation of the Characterizing Freezing of gait questionnaire: implementation of the TRAPD process
Source: J Patient Rep Outcomes. 2025 Nov 14;9:132. doi: 10.1186/s41687-025-00967-1 (PMC12618777; doi:10.1186/s41687-025-00967-1)
Supplement: Supplementary file 2 — Supplementary Material 2 - C-FOG- D / C1 Self-Assessment [file 41687_2025_967_MOESM4_ESM.pdf]

GERMAN TRANSLATION OF THE CHARACTERIZING FREEZING OF GAIT QUESTIONNAIRE: IMPLEMENTATION OF THE TRAPD PROCESS

Supplementary Material

Self-designed C1 Self-Assessment Questionnaire for Recruitment for Review Stage

Demographics:

- Is English your first language (the language you acquired during early childhood)?
- Self-reported gender? – Open Question
- Age group? 18-30, 30-40, 40-50, 50-60, over 61?

Instructions: Answer each question honestly based on your current language skills.

Overall Oral Production:	
Can you comfortably explain complex subjects in detail, including subtopics and supporting points?	Yes = 1 / No = 0
Do you struggle to express yourself fluently when discussing complex topics?	Yes = 0 / No = 1
Overall Reading Comprehension:	
Can you understand lengthy and complex texts, even if they require rereading in some parts?	Yes = 1 / No = 0
Are you comfortable reading a variety of materials, including literature, news articles, and academic publications (with potential rereading)?	Yes = 1 / No = 0
Co-operation:	
Can you effectively build upon the contributions of others during discussions and conversations?	Yes = 1 / No = 0
Do you sometimes find it difficult to integrate your ideas smoothly with those presented by others?	Yes = 0 / No = 1
Sum	Max. 6 Points
